# Curation of an international drug proprietary names dataset

**DOI:** 10.1016/j.dib.2021.107701

**Published:** 2021-12-10

**Authors:** Mohammad A. Khaleel, Amer Hayat Khan, S.M. Sheikh Ghadzi, Sami Alshakhshir

**Affiliations:** a Discipline of Clinical Pharmacy, School of Pharmaceutical Sciences, Universiti Sains Malaysia, Penang 11800, Malaysia; b Discipline of Clinical Pharmacy, School of Pharmacy, Aqaba University of Technology, Jordan

**Keywords:** Drug list, Drug dataset, Drug database, Drug lexicon, Mapping to RxNorm, Proprietary drug name, Drug trade name, Drug active ingredient name

## Abstract

A drug dataset containing international proprietary names is essential for researchers investigating different drugs from different countries worldwide. However, many websites on the internet offer free access for a single drug searching service to identify international drug trade names, but not for a list of drugs to be searched and identified. Therefore, it will be problematic if the researcher has a list of hundreds or thousands of drug trade names to be identified. In this project, we have created an International Drug Dictionary (IDD) by curating collected drug lists from open access websites belonging to official drug regulatory agencies, official healthcare systems, or recognized scientific bodies from 44 countries around the world in addition to the European public assessment reports (EPAR) and the DRUGBANK vocabulary published in the public domain. Researchers interested in pharmacovigilance, pharmacoepidemiology, or pharmacoeconomics can benefit from this dataset, especially when identifying lists of proprietary drug names, particularly of multi-national origin.

To enhance its adaptability, we also mapped the IDD to the standardized drug vocabulary RxNorm. The IDD can also be used as a tool for mapping international drug trade names to RxNorm. Each drug entity in the IDD mapped to a unique identification number for each entity called Atom Unique Identifier (RXAUI) from RxNorm.

**Specifications Table**

**Table:** 

Subject	Health informatics
Specific subject area	Mapping of proprietary drug names to standardized active ingredient names
Type of data	Table microsoft access database
How data were acquired	The original datasets are freely available from the official websites of: National Agency of Drugs and Medical Devices (ALBANIA); Pharmaceutical Benefits Scheme (PBS), (AUSTRALIA); Austrian Federal Office for Safety in Health Care; National Health Regulatory Authority (BAHRAIN); Federal Agency for Medicines and Health Products (FAMHP), (BELGIUM); Agency for Medicinal Products and Medical Devices of Bosnia andHerzegovina; Brazilian Health Regulatory Agency (Anvisa); Health Canada; Colombia Open Data portal; The Agency for Medicinal Products and Medical Devices (HALMED),(CROATIA); Regulatory Authority of Medicines, Equipment and Medical Devicesof the Republic of Cuba (CECMED); DRUGBANK; The European Medicines Agency (EMA); Finnish Medicines Agency (Fimea); The National Agency for the Safety of Medicines and Health Products(ANSM), (FRANCE); Drug Office, Department of Health (HONG KONG); National Institute of Pharmacy and Nutrition (HUNGARY); Icelandic Medicines Agency; Food and Drug Administration (IRAN); Health Products Regulatory Authority (HPRA), (IRELAND); Pharmaceuticals and Medical Devices Agency (JAPAN); Jordan Food and Drug Administration; Medicinal Product Register of Latvia; State Drug Control Service (VAPRIS), (LITHUANIA);Ministry of Health (MALDIVES); Government of Mexico Portal - gob.mx; Namibia Medicines Regulatory Council; New Zealand Medicines and Medical Devices Safety Authority (Medsafe); The Norwegian Medicines Agency, NoMA medicine database; Register of Medicinal Products (POLAND); The Portuguese Database of Medicines for Human Use (Informed); Ministry of Public Health (QATAR); Index Of Medicinal Products for Human Use, National Agency forMedicines And Medical Devices of Romania; Saudi Food and Drug Administration; State Institute for Drug Control (SLOVAKIA); Central Database of Medicinal Products (Centralna baza zdravil),(SLOVANIA); South African Medicine Price Registry; Medicine Online Information Centre of AEMPS CIMA (SPAIN); National Medicines Regulatory Authority (NMRA), (SRI LANKA); Swedish Medical Products Agency; SWISS Agency for Therapeutic Products (SWISSMEDIC); Tanzania Medicines & Medical Devices authority; Turkey Pharmaceuticals and Medical Devices Agency; National Drug Authority (UGANDA); NHS Digital, TRUDE (UK); FDA (USA).
Data format	Raw processed
Parameters for data collection	The data source for trade names should be an official website in each country and be freely available to the public. The trade name list must contain either the active ingredient name or the ATC code of drugs in that list. Drug names should be in a Latin-based alphabet. The DRUGBANK [1] vocabulary was used as a source of synonyms and alternative names rather than as a source of trade names.
Description of data collection	We downloaded the datasets from the official websites of drug authorities or agencies in 44 countries in addition to the European public assessment reports (EPAR) and the DRUGBANK vocabulary.
Data source location	Primary Data Sources, in the same order as relevant agencies mentioned above: http://akbpm.gov.al/ https://www.pbs.gov.au/pbs/home https://aspregister.basg.gv.at/aspregister/faces/aspregister.jspx https://www.nhra.bh/ https://www.fagg.be/nl/items/gegevensbank_vergunde_geneesmiddelen http://www.almbih.gov.ba/lijekovi/ https://dados.gov.br/dataset/medicamentos-registrados-no-brasil https://www.canada.ca/en/health-canada/services/drugs-health-products/drug-products/drug-product-database.html https://www.minsalud.gov.co/sites/rid/Lists/BibliotecaDigital/RIDE/VS/MET/Informacion-estandarizada-cargue-inicial.zip https://www.halmed.hr/en/Lijekovi/Baza-lijekova/#rezultati https://servicio.cecmed.cu/sicecmed/libroRegistroMedicamento/index# https://go.drugbank.com/releases/latest#open-data https://www.ema.europa.eu/sites/default/files/Medicines_output_european_public_assessment_reports.xlsx https://www.fimea.fi/laakehaut_ja_luettelot/perusrekisteri http://agence-prd.ansm.sante.fr/php/ecodex/telecharger/telecharger.php https://www.drugoffice.gov.hk/eps/do/en/consumer/home.html https://ogyei.gov.hu/drug_database https://www.lyfjastofnun.is/utgefid-efni/listar/ https://www.fda.gov.ir/WHAttachments/3a/3a5227ae-772a-413d-8855–3c15fed743f1.xls http://www.hpra.ie/img/uploaded/swedocuments/latestHMlist.pdf https://www.pmda.go.jp/english/review-services/reviews/approved-information/drugs/0002.html http://www.jfda.jo/Pages/viewpage.aspx?pageID=175 https://www.zva.gov.lv/zvais/zalu-registrs/?&lang=en https://vapris.vvkt.lt/vvkt-web/public/medications http://health.gov.mv/Downloads https://www.gob.mx/cms/uploads/attachment/file/534996/Listado_de_medicamentos_de_Referencia_.pdf https://nmrc.gov.na/medicine-register https://www.medsafe.govt.nz/regulatory/DbSearch.asp https://www.legemiddelsok.no/ https://rejestrymedyczne.ezdrowie.gov.pl/rpl/search/public https://extranet.infarmed.pt/INFOMED-fo/ https://www.moph.gov.qa/english/derpartments/policyaffairs/pdc/regnpricing/Pages/default.aspx https://www.anm.ro/nomenclator/medicamente https://www.sfda.gov.sa/en/drugs-list https://www.sukl.sk/verejne/Zoznam_liekov/zoznam_liekov.zip http://www.cbz.si/cbz/bazazdr2.nsf/Search/$searchForm?SearchView http://www.mpr.gov.za/PublishedDocuments.aspx#DocCatId=21 http://cima.aemps.es/cima/publico/nomenclator.html https://nmra.gov.lk/index.php?lang=en https://www.lakemedelsverket.se/sv/sok-lakemedelsfakta?activeTab=1 https://www.SWITZERLANDmedic.ch/SWITZERLANDmedic/en/home/services/listen_neu.html#319575900
	https://imis2.tmda.go.tz/portal/#/public/registered-medicines https://www.titck.gov.tr/dinamikmodul/85 https://www.nda.or.ug/drug-register-downloads/ https://isd.digital.nhs.uk/trud3/user/guest/group/0/pack/6/subpack/24/releases https://www.fda.gov/drugs/drug-approvals-and-databases/drugsfda-data-files https://www.fda.gov/drugs/drug-approvals-and-databases/national-drug-code-directory
Data accessibility	For the IDD dataset Repository name: Mendeley Data Data identification number: 10.17632/9nmgzttxhm.1 Direct URL to data: https://data.mendeley.com/datasets/9nmgzttxhm/1 For the code [2] used to generate the IDD Repository name: Zenodo Data identification number: 10.5281/zenodo.5711846 URL to the code: https://doi.org/10.5281/zenodo.5711846

**Value of the Data**

• Being a combined and cured dataset from 46 sources globally makes the IDD suitable for identifying most drug proprietary names globally.
• Researchers interested in pharmacovigilance, pharmacoepidemiology, or pharmacoeconomics can benefit from this dataset, especially when identifying lists of proprietary drug names.
• The IDD provides a tool for mapping proprietary drug names to the active ingredient level in the standard vocabulary RxNorm [3].
• The introduction of the cleaned trade names field would enhance matching with other drug lists.
• The added English translation for active ingredients to some sources would also enhance matching.

## 1 Data Description

The data provided is composed of one Excel sheet file and one Microsoft Access database file. The excel sheet contains a single table of the whole dataset of drug names in the IDD, mapped to the ingredient level in RxNorm, as shown in Table 1.

**Table 1:** Preview of IDD in the Excel sheet format.

DRUGNAME	RXAUI	RXCUI	STR	SAB	TTY	CODE
SULFACEL	10285070	82101	Sulfacetamide Sodium	USP	IN	4NRT660KJQ
TENAXUM	529502	55679	rilmenidine	RXNORM	IN	55679
ALPRAZOLAM TABLETS 0.5MG	12251530	596	alprazolam	RXNORM	IN	596
RUPILIP	1386783	301542	rosuvastatin	RXNORM	IN	301542
NIMODIPINE NORDIC	12253925	7426	nimodipine	RXNORM	IN	7426
Nilol	12353899	392475	atenolol / nifedipine	RXNORM	MIN	392475
NETILDEX	5479653	1430042	dexamethasone and antiinfectives	ATC	IN	S01CA01

*Abbreviations:* RXAUI, the RxNorm atom identifier; RXCUI, the RxNorm concept identifier; STR, String as in RxNorm; SAB, Source abbreviation as in RxNorm; TTY, Term type in the source vocabulary as in RxNorm; CODE, the source asserted identifier as in RxNorm.

The Access database file helps users with limited technical background retrieve active ingredient names from lists containing brand names. The user of the access database can search for a single drug or do a batch matching by importing an Excel datasheet of the drugs of interest into the access database through a simplified user-friendly interface. Results of the list matching can be viewed inside the Access database instantly, or it can be exported into Excel or PDF file format.

However, since this dataset was not manually reviewed in full, we do not recommend using it to make any clinical decision.

## 2 Experimental Design, Materials and Methods

Drug lists downloaded from the official websites and drug names were extracted from its original lists (i.e., Excel sheets, Access database, CSV, XML, text and pdf files), then merged into one larger table or database of proprietary drug names with their relevant alternative names or active ingredient names.

All individual source tables (i.e., text, CSV, and Excel files) were imported directly into a SQL server database. Those datasets composed of more than one table were first imported into the Microsoft Access database to make the needed relationships between tables and export one unified table into the SQL server database. If the source vocabulary was presented as a pdf file, then first the pdf file was converted into an excel file, the excel file then checked for integrity, then imported into the SQL server database. Only one dataset wherein XML format (NHSBSA md+d, UK) and for that specific dataset, the relevant excel files were extracted through the official dm+d XML transformation tool [4]. Tables were imported into the Microsoft Access database to establish a proper relationship between the dataset files, and one table was extracted then exported to the SQL Server. Then all tables from all sources were merged using SQL server queries into one larger table by selecting distinct rows from each source. Then, multistep SQL server queries were made for each source dataset separately to create alternate drug names that are shorter and cleaner than the source names as needed (i.e., removing dosage form, strength, unite of strength, or manufacturer name). The original drug entries were kept without any processing, except for very limited removal of trailing spaces and some unexplained trailing symbols from the original fields.

If the source drug list was not in English and did not contain ATC codes for drug names, then relevant active ingredient names from the source list were translated by exporting the list to a Google sheet, then performing a translation to English through Google API translation on that sheet, and finally updating the source drug list with the English translation as an alternative name for active ingredients. The DRUGBANK vocabulary was added to the IDD as it contains synonym terms for active ingredients. That would enhance the matching and linking between drug names, especially when mapping active ingredients spelled differently by different languages. To enhance the adaptability of the IDD, we mapped drug names to the standardized drug vocabulary RxNorm. All the drug name fields in each source dataset (i.e., trade name, active ingredient, other alternative names if present, cleaned trade names, cleaned active ingredient names) were matched separately to STR column (drug string) in RXNCONSO table from RxNorm to map each drug entity in the IDD to a specific Atom Unique Identifier (RXAUI) from RxNorm. Then, we used the available ATC codes from the source drug lists to match with ATC codes from RxNorm to further identify drug names that could not be matched directly to STR field in RxNorm. Only successfully mapped drug names to RxNorm were included in the final set of the dictionary.

Finally, each row in the dataset was divided into multiple rows so that each new row consists of only one drug field (i.e., trade name, cleaned trade name, active ingredient, cleaned active ingredient, or alternative name) in addition to the RxNorm attributes (i.e., RXAUI, RXCUI, STR, SAB, TTY, and CODE) as in the original row.

To verify the integrity of the IDD, we randomly selected 50 drug names and manually verified the trade names and the linked active ingredients from RxNorm with other attributes.

## Ethics Statement

The project does not involve any type of human studies, animal studies, or data gathered using social media, So ethical approval has not been sought. We can confirm that this manuscript adheres to ethics in publishing standards.

## CRediT authorship contribution statement

**Mohammad A. Khaleel:** Conceptualization, Methodology, Resources, Data curation, Software, Validation, Writing – original draft, Writing – review & editing. **Amer Hayat Khan:** Supervision, Investigation, Writing – review & editing. **S.M. Sheikh Ghadzi:** Investigation, Writing – review & editing. **Sami Alshakhshir:** Resources, Validation.

## Declaration of Competing Interest

The authors declare that they have no known competing financial interests or personal relationships which have or could be perceived to have influenced the work reported in this article.
